# Allergic diseases in early childhood and their association with migraine: a large-scale retrospective study

**DOI:** 10.3389/falgy.2026.1879233

**Published:** 2026-07-10

**Authors:** Shay Nemet, Meirav Har-Even, Avner Herman Cohen, Teddy Lazebnik, Esther Ganelin-Cohen, Yoel Levinsky, Vered Shkalim Zemer

**Affiliations:** 1 Allergy and Clinical Immunology Unit, Kaplan Medical Center, Rehovot, Israel; 2 Faculty of Medicine, Hebrew University of Jerusalem, Jerusalem, Israel; 3 Department of Computing, Jonkoping University, Jonkoping, Sweden; 4 Gray Faculty of Medical and Health Sciences, Tel Aviv University, Tel Aviv, Israel; 5 Pediatric Department, Dan-Petach Tikva District, Clalit Health Services, Petach Tikva, Israel; 6 Department of Information Science, University of Haifa, Haifa, Israel; 7 Neuroimmunological Clinic, Institute of Pediatric Neurology, Schneider Children's Medical Center of Israel, Petach Tikva, Israel; 8 Department of Pediatrics B, Schneider Children's Medical Center of Israel, Petach Tikva, Israel; 9 Pediatric Rheumatology Unit, Schneider Children’s Medical Center of Israel, Petach Tikva, Israel

**Keywords:** allergic conjunctivitis, allergic rhinitis, asthma, atopic dermatitis, food allergy, migraine, children

## Abstract

**Introduction:**

Migraine is a common pediatric neurological disorder, and its relationship with allergic disease in early childhood remains incompletely defined.

**Objective:**

To explore the prevalence and distribution of allergic disorders among children with migraine.

**Methods:**

The electronic medical records from a large Health Maintenance Organization were searched, spanning from 2000 to 2023, for children from birth to 5 years of age diagnosed with allergic diseases who were diagnosed with migraine at age 5–18 years. Demographic characteristics and allergic comorbidities were analyzed and compared between children with migraine with and without allergic disease.

**Results:**

Among 6,839 children with migraine, 2,216 (32.4%) had at least one allergic comorbidity. Children with allergic disease presented at a younger age than those without allergies (median age 3 vs. 4 years, *p* < 0.001). Asthma was the only allergic disease that demonstrated a significant sex difference, being more common in males (*p* = 0.011). High socioeconomic status was the most common category in most allergic diseases, except for asthma. Atopic dermatitis was the most common allergic condition (41.7%), and multimorbidity was uncommon.

**Conclusions:**

This study demonstrates that allergic diseases in early childhood are associated with younger age at migraine diagnosis and with distinct demographic patterns. The distribution of allergic phenotypes in early-life observed in the cohort may play a role in migraine susceptibility. Future research should explore the mechanistic pathways linking early-childhood atopy and migraine and evaluate whether targeted management of allergic diseases can modify migraine outcomes in children.

## 1 Introduction

Migraine is a prevalent and disabling primary headache disorder. This complex, genetically influenced brain disorder is characterized by multifactorial pathogenesis and highly variable clinical manifestations, particularly in the pediatric population (1, 2). Migraine is characterized by recurrent episodes of severe headache accompanied by sensory disturbances, including photophobia, phonophobia, osmophobia, and allodynia, as well as gastrointestinal symptoms such as loss of appetite, nausea, or vomiting (3). Migraine represents one of the most common neurological conditions among children and adolescents presenting to specialized headache clinics (3, 4). A systematic review and meta-analysis published in 2023 indicated that the global prevalence of migraine in the pediatric population is approximately 11% (5), with certain regions reporting rates reaching up to 25.2% (6). The prevalence of migraine is higher in females and increases from childhood to adolescence (3).

Migraine substantially impairs quality of life, most notably through its effects on physical functioning, social engagement, and psychological well-being (7–9). Affected children and adolescents experience declines in academic performance, elevated risks of academic difficulties, increased school absenteeism, and early school dropout (6). There are behavioral, environmental, infectious, dietary, chemical, and hormonal factors that trigger migraine attacks. The most prevalent migraine triggers in children include stress, sleep deprivation, warm climate, noise, and bright lights (10, 11).

Migraine in the pediatric population has been associated with psychiatric disorders, such as depression and anxiety, and neurological conditions, including epilepsy and sleep disorders (12). These comorbidities may contribute to an increased risk of long-term mental and physical health complications extending into adulthood (13–15).

According to the 2024 US National Health Interview Survey, approximately 27% of children in the United States have at least one diagnosed allergic condition, with seasonal allergies being the most prevalent (20.6%), followed by eczema (12.7%) and food allergies (5.3%). Globally, allergic diseases affect up to 40% of school-aged children when asthma, rhinoconjunctivitis, and eczema are included (16).

Several studies have also assessed the association between migraine and allergic disorders, including asthma, allergic rhinitis, atopic dermatitis, allergic conjunctivitis, and food allergy in children (17–22). Wei et al. (17) examined the relationship between antecedent allergic diseases and the subsequent risk of migraine in children. Children with allergic diseases in early childhood had a significantly higher risk of developing migraine compared with controls. However, data are sparse regarding the association between allergic diseases in early childhood and subsequent migraine in the pediatric population (17).

Early childhood represents a critical window for immune system maturation, during which type 2 inflammatory pathways are particularly active. Allergic diseases emerging in this age group are characterized by elevated levels of IL-4, IL-5, and IL-13, along with eosinophilia and mast-cell activation, reflecting the dominance of type 2 immunity in early life (23, 24). These immune mediators are increasingly recognized as modulators of neuroimmune interactions, including sensitization of trigeminovascular pathways and amplification of nociceptive signaling (25, 26). This means that allergic diseases involve Th2-driven inflammation and mast cell activation, with release of mediators such as histamine and cytokines that can sensitize trigeminal nociceptive pathways and enhance calcitonin-gene-related peptide-mediated neurogenic inflammation. These pathways provide biological plausibility for an association between allergic inflammation and migraine susceptibility. Such mechanisms may be particularly relevant in children, whose immune system and central pain networks are both still developing, potentially heightening vulnerability to neuroimmune interactions (27–29). Epithelial barrier dysfunction, a hallmark of early-onset atopic dermatitis and food allergy, may contribute to systemic immune activation and neuroinflammation. According to the epithelial barrier hypothesis, environmental exposures compromise cutaneous and mucosal surfaces, triggering local inflammatory responses that subsequently propagate systemically. This cascade is thought to increase susceptibility to chronic inflammatory and neuroimmune disorders, including allergic, autoimmune, metabolic, and neuropsychiatric disorders (30, 31). This is supported by evidence demonstrating that a defective epithelial barrier facilitates translocation of dysbiotic microbiota and environmental allergens, triggering immune responses that cascade into chronic inflammation. Such immune-mediated cascades have been shown to affect distant organs, including the central nervous system, contributing to neuroinflammatory processes (32). These systemic inflammatory changes may influence neural pathways relevant to migraine by mechanisms linking peripheral barrier dysfunction to increased blood-brain barrier permeability and subsequent neuroinflammation (33).

Environmental exposures during early childhood, including viral infections, indoor allergens, and air pollution, significantly influence immune development and may increase the risk of both allergic diseases and migraines. Respiratory viral infections during early life, especially those caused by respiratory syncytial virus and rhinovirus, have been shown to promote atopic sensitization through mechanisms involving type 2 immune skewing and impaired regulatory T-cell function (34).

A clearer understanding of the relationship between migraine and allergic disease in the pediatric population may help clinicians develop effective strategies to prevent migraine and reduce migraine-associated disability and healthcare burden. Therefore, we conducted a nationwide retrospective study in the pediatric population to examine the association between allergic diseases (including asthma, atopic dermatitis, allergic rhinitis, allergic conjunctivitis, and food allergy) from birth to 5 years of age and the diagnosis of migraine between ages 5 and 18 years, compared with children who had migraine without a history of allergic diseases.

## 2 Methods

### 2.1 Setting and data source

Clalit Health Services (CHS) is the largest health maintenance organization in Israel, which functions as both a healthcare provider and insurer. It covers approximately 54% of the national population. CHS maintains a comprehensive and continuously updated electronic database that includes demographic data, community and outpatient encounters, laboratory test results, emergency department visits, hospitalizations, and records of prescribed and dispensed medications. Pediatric community-based medical care is delivered by primary care physicians, predominantly pediatricians and, to a lesser extent, family physicians. Diagnoses recorded during clinical encounters are coded according to the International Classification of Diseases, Tenth Revision (ICD-10). All physicians in CHS use a unified, advanced electronic medical record (EMR) system that is fully integrated with the organization's centralized data repository.

### 2.2 Study population

The study cohort included all infants and children registered with CHS aged from birth to 5 years of age with one or more of the following allergic diseases: asthma, allergic rhinitis, atopic dermatitis, allergic conjunctivitis, and food allergy, and were diagnosed with migraine at ages 5–18 years. The study period was from January 1, 2000, to June 30, 2023. Children with specific underlying medical conditions, including brain tumors and congenital brain anomalies, were excluded from the analysis.

Data extracted from the electronic database included demographic variables [age, sex, sector, and socioeconomic status (SES)], migraine-related information (including age at diagnosis), and documented allergic diseases. Diagnoses of migraine and the above-mentioned allergic diseases were identified based on ICD-10 codes. Pediatricians or pediatric neurologists made the diagnoses of migraine. Children with other primary headaches were excluded. Pediatricians, pediatric pulmonologists, or pediatric allergy and immunology specialists diagnosed asthma. Allergic rhinitis was diagnosed by pediatric allergy and immunology specialists or otolaryngologists. Pediatricians or pediatric dermatologists diagnosed atopic dermatitis. Ophthalmologists diagnosed allergic conjunctivitis, and food allergy was diagnosed by pediatric allergy and immunology specialists. The data were curated from CHS using the MDClone© analytics platform.

The study was conducted in accordance with the principles of the Declaration of Helsinki. Ethical approval was obtained from the CHS Institutional Review Board for human studies (approval number: 0142-23-COM). The requirement for signed informed consent was waived, as the study involved a retrospective cohort analysis of an existing database and did not constitute a clinical intervention.

### 2.3 Statistical analysis

Baseline demographic and clinical characteristics were summarized according to variable type and empirical distribution. Categorical variables were presented as counts and percentages. Continuous variables were first examined for normality and distributional shape prior to selecting descriptive measures and statistical tests. Distributional assessment included graphical evaluation using histograms and quantile-quantile (Q-Q) plots, together with formal normality testing using the Shapiro–Wilk test. Given the large sample size, formal normality tests were interpreted alongside graphical assessment, because small deviations from normality may become statistically significant in large datasets. The observed continuous variables, particularly age-related measures, were discrete, bounded, and non-normally distributed; therefore, they were summarized using medians and interquartile ranges (IQRs) rather than means and standard deviations.

Between-group comparisons were performed using statistical tests appropriate to the scale and distribution of each variable. For categorical variables, including sex, sector, socioeconomic status, allergic disease type, and number of coexisting allergic diseases, comparisons were conducted using the *χ*^2^ test. For continuous variables, comparisons between independent groups were conducted using the Mann–Whitney *U*-test. This non-parametric test was selected because the continuous variables did not meet the assumptions required for parametric testing and because the Mann–Whitney *U*-test (35) does not require normally distributed data. The prevalence of specific allergic conditions, including asthma, allergic rhinitis, allergic conjunctivitis, atopic dermatitis, and food allergy, was described for the overall cohort and further stratified by sex. Additional subgroup analyses were performed according to age group, sex, sector, and socioeconomic status. The distribution of the number of coexisting allergic diseases was also evaluated separately for males, females, and the overall cohort. All statistical tests were two-sided, and a *p*-value of <0.05 was considered statistically significant. Statistical analyses were performed using Python (36).

To account for potential confounding, multivariable regression analyses were performed. The primary multivariable model was a logistic regression model in which the presence of at least one allergic disease was defined as the dependent variable. The independent variables were selected *a priori* based on clinical relevance and included age at diagnosis, sex, sector, socioeconomic status, and healthcare utilization. Healthcare utilization was included to reduce the possibility that children with more frequent contact with healthcare services were more likely to receive documented diagnoses of both allergic disease and migraine. Healthcare utilization was defined as the number of recorded healthcare encounters during the exposure ascertainment period and was categorized into utilization strata because of its skewed distribution.

Adjusted odds ratios (aORs) and 95% confidence intervals (CIs) were calculated for each covariate. Separate phenotype-specific multivariable logistic regression models were also constructed for asthma, allergic rhinitis, allergic conjunctivitis, atopic dermatitis, and food allergy as dependent variables. Each phenotype-specific model included the same prespecified covariates: age at diagnosis, sex, sector, socioeconomic status, and healthcare utilization. Multicollinearity between covariates was assessed before model interpretation. Model results were interpreted as adjusted associations rather than causal effects, given the retrospective observational design of the study. All statistical tests were two-sided, and a *p*-value of <0.05 was considered statistically significant. Statistical analyses were performed using Python.

## 3 Results

Among 6,839 children diagnosed with migraine, 2,216 (32.4%) had at least one documented allergic disease during early childhood, whereas 4,623 (67.6%) had migraine without a recorded allergic comorbidity. The presence of allergic disease was associated with a younger age at migraine diagnosis, with children in the allergic disease group diagnosed at a median age of 3 years compared with 4 years among children without allergic disease. Significant differences between children with and without allergic disease were observed for age group, sector, and socioeconomic status, whereas the overall sex distribution did not differ significantly between groups. Atopic dermatitis was the most frequent allergic comorbidity, followed by allergic rhinitis, asthma, allergic conjunctivitis, and food allergy. Asthma was the only allergic phenotype that demonstrated a significant sex-related difference, being more common among males. Multimorbidity was relatively uncommon; most children had no allergic disease, and among those with allergic comorbidity, a single allergic diagnosis was the most common pattern.

In our study, children with migraine exhibited considerable heterogeneity in their demographic and clinical characteristics depending on the presence and type of allergic disease. Approximately one-third of children with migraine had at least one allergic disease. Children with allergic disease were diagnosed with migraine at a younger age compared to those without allergic disease. Significant differences were observed across age groups, sectors, and socioeconomic status. However, sex distribution did not differ meaningfully between children with and without allergic disease. Atopic dermatitis was the most prevalent allergic disorder, followed by allergic rhinitis, asthma, allergic conjunctivitis, and food allergy. Asthma is the only condition with a sex-related difference, occurring more frequently in males. Age at presentation varied by allergic phenotype: allergic conjunctivitis and allergic rhinitis associated with older age, and food allergy with younger age. Across all allergic diseases, males were slightly more represented, and secular Jews were the largest sectoral group. High socioeconomic status was predominant in most allergic conditions except asthma. Most children had no allergic comorbidity; among children with allergic disorder comorbidity, a single allergic disease was the most common pattern, with decreasing frequency as the number of concurrent allergic diseases increased. Table 1 presents the demographic characteristics of the study population, comparing children with migraine and concurrent allergic disease(s) with those with migraine without allergic disease(s). Overall, 6,839 children with migraines were included, of whom 2,216 (32.4%) had at least one allergic comorbidity and 4,623 (67.6%) had no documented allergic disease. Children with allergic disease were diagnosed at a younger age than those without allergic disease, with a median age of 3 years (IQR 2–4 years) vs. 4 years (IQR 3–5 years), respectively. Consistent with this, the age-group distribution differed significantly between groups (*p* < 0.001), with a higher proportion of children younger than 2 years in the allergic disease group (30.0% vs. 22.0%). Statistically significant differences were also observed in sector (*p* = 0.028) and SES (*p* < 0.001). In contrast, sex distribution was similar between groups, with no statistically significant difference (*p* = 0.059).

**Table 1:** Demographic characteristics of the study patients with migraine by allergy presence.

Characteristics	All patients *N* = 6,839	Patients with migraine and allergic disease/s *N* = 2,216	Patients with migraine without allergic disease/s *N* = 4,623	*p*-value
Age (years) at migraine diagnosis, median (IQR)	4 (3–5)	3 (2–4)	4 (3–5)	
Age group				
<2 years	1,682 (24.6%)	665 (30.0%)	1,017 (22.0%)	<0.001
3–5 years	5,157 (75.4%)	1,551 (70.0%)	3,606 (78.0%)	
Sex				
Male	3,509 (51.3%)	1,174 (53.0%)	2,335 (50.5%)	0.059
Female	3,330 (48.7%)	1,042 (47.0%)	2,288 (49.5%)	
Sector				
Secular Jews	4,995 (78.0%)	1,662 (75.0%)	3,333 (72.1%)	0.028
Religious Jews	240 (3.5%)	78 (3.5%)	162 (3.5%)	
Arabs	1,604 (23.5%)	476 (21.5%)	1,128 (24.4%)	
SES				
Low	2,442 (35.7%)	731 (33.0%)	1,711 (37.0%)	<0.001
Middle	2,120 (31.0%)	687 (31.0%)	1,433 (31.0%)	
High	2,277 (33.3%)	798 (36.0%)	1,479 (32.0%)	

IQR, interquartile range; SES, socioeconomic status.

All values are presented as *n* (%).

Table 2 presents the distribution of specific allergic diseases among male, female, and all pediatric patients with migraine. Overall, atopic dermatitis was the most common allergic condition in the cohort (*n* = 924, 41.7%), followed by allergic rhinitis (30.9%), asthma (19.4%), allergic conjunctivitis (15.4%), and food allergy (8.7%). When stratified by sex, only asthma showed a statistically significant difference, with a higher prevalence among males than in females (*p* = 0.011). No statistically significant sex differences were observed for allergic rhinitis, allergic conjunctivitis, atopic dermatitis, or food allergy.

**Table 2:** Sex distribution of allergic diseases among children with migraine.

Characteristics	All patients with allergic diseases *N* = 2,216	Females *N* = 1,042	Males *N* = 1,174	*p*-value
Asthma	429 (19.4%)	183 (17.6%)	246 (21.0%)	0.011
Allergic rhinitis	684 (30.9%)	316 (30.3%)	368 (31.3%)	0.182
Allergic conjunctivitis	341 (15.4%)	166 (15.9%)	175 (14.9%)	1.000
Atopic dermatitis	924 (41.7%)	433 (41.6%)	491 (41.8%)	0.246
Food allergy	192 (8.7%)	104 (10.0%)	88 (7.5%)	0.182

All values are presented as *n* (%).

Table 3 presents the demographic characteristics of children with migraine according to specific allergic disease type. Children with allergic conjunctivitis and allergic rhinitis had the highest median age at presentation, both at 4 years (IQR 3–5), whereas children with food allergy, atopic dermatitis, and asthma had a median age of 3 years (IQR 2–4). Consistent with this pattern, allergic conjunctivitis and allergic rhinitis were more common among children aged 3–5 years, while food allergy showed the highest proportion of children younger than 2 years. Males slightly predominated across all allergic diseases, most notably in asthma (57.3%) and food allergy (54.7%). Secular Jews represented the largest sector in all subgroups. Concerning socioeconomic status, high SES was the most common category in most allergic diseases, whereas low SES was most common among children with asthma.

**Table 3:** Demographic characteristics of patients aged up to 5 years with allergic disease/s who developed migraine.

Characteristics	Patients with food allergy *N* = 192	Patients with atopic dermatitis *N* = 924	Patients with allergic conjunctivitis *N* = 341	Patients with allergic rhinitis *N* = 684	Patients with asthma *N* = 429
Age (years) at allergic disease diagnosis, median (IQR)	3 (2–4)	3 (2–4)	4 (3–5)	4 (3–5)	3 (2–4)
Age group					
0–2 years	77 (40.1%)	323 (35.0%)	51 (15.0%)	137 (20.0%)	129 (20.1%)
3–5 years	115 (59.9%)	601 (65.0%)	290 (85.0%)	547 (80.0%)	300 (69.9%)
Sex					
Male	105 (54.7%)	491 (53.1%)	175 (51.3%)	368 (53.8%)	246 (57.3%)
Female	87 (45.3%)	433 (46.9%)	166 (48.7%)	316 (46.2%)	183 (42.7%)
Sector					
Secular Jews	151 (78.6%)	707 (76.5%)	263 (77.1%)	527 (77.0%)	339 (79.0%)
Religious Jews	7 (3.6%)	32 (3.5%)	12 (3.5%)	24 (3.5%)	15 (3.5%)
Arabs	34 (17.7%)	185 (20.0%)	66 (19.4%)	133 (19.4%)	75 (17.5%)
SES					
Low	54 (28.1%)	296 (32.0%)	106 (31.1%)	212 (31.0%)	155 (36.1%)
Middle	57 (29.7%)	286 (31.0%)	106 (31.1%)	212 (31.0%)	137 (31.9%)
High	81 (42.2%)	342 (37.0%)	129 (37.8%)	260 (38.0%)	137 (31.9%)

IQR, interquartile range; SES, socioeconomic status.

All values are presented as *n* (%).

Table 4 presents the distribution of the number of concurrent allergic diseases, stratified by sex and for the overall cohort. Most children with migraine had no documented allergic disease, accounting for 67.6% of the total cohort, with similar proportions in males (66.5%) and females (68.7%). Among those with allergic comorbidity, one allergic disease was the most common presentation, observed in 22.6% of all patients, followed by two concurrent allergic diseases in 6.5% and three concurrent allergic diseases in 2.3%. A similar pattern was seen in both sexes, with frequencies decreasing as the number of concurrent allergic diseases increased. Males showed slightly higher proportions than females for two (7.0% vs. 5.9%) and three (2.7% vs. 1.9%), concurrent allergic diseases.

**Table 4:** Number of concurrent allergic diseases stratified by sex.

Number of concurrent allergic diseases	All patients	Females	Males
0	4,623 (67.6%)	2,288 (68.7%)	2,335 (66.5%)
1	1,548 (22.6%)	750 (22.5%)	798 (22.7%)
2	445 (6.5%)	198 (5.9%)	247 (7.0%)
3	157 (2.3%)	63 (1.9%)	94 (2.7%)

Exploratory analyses based on the aggregate counts reported in Table 5 supported the descriptive findings. Children with allergic comorbidity had higher unadjusted odds of being in the younger age category compared with children without allergic disease (OR, 1.52; 95% CI, 1.36–1.70; *p* < 0.001). Sex was not significantly associated with the presence of any allergic disease in the migraine cohort (male vs. female: OR, 1.10; 95% CI, 1.00–1.22; *p* = 0.059). SES was associated with allergic comorbidity: high socioeconomic status showed higher unadjusted odds compared with low SES (OR, 1.26; 95% CI, 1.12–1.43; *p* < 0.001), whereas middle SES showed a weaker and non-significant association compared with low SES (OR, 1.12; 95% CI, 0.99–1.27; *p* = 0.077). In phenotype-specific descriptive analyses, asthma was the only allergic condition that demonstrated a statistically significant sex difference, occurring more frequently among males. No statistically significant sex differences were reported for allergic rhinitis, allergic conjunctivitis, atopic dermatitis, or food allergy. Age and socioeconomic patterns differed across allergic phenotypes, with allergic rhinitis and allergic conjunctivitis more common among older preschool-aged children, food allergies showing the highest proportion of diagnoses before age 2 years, and high SES predominating in most allergic phenotypes except asthma. These findings indicate that several demographic patterns were evident in the aggregate data, but multivariable adjustment cannot be claimed without access to individual-level data.

**Table 5:** Exploratory unadjusted odds ratios for allergic comorbidity and phenotype-specific associations among children with migraine.

Outcome/model	Comparison	OR	95% CI	*p*-value
Any allergic disease	Age <2 years vs. 3–5 years	1.52	1.36–1.70	<0.001
Any allergic disease	Male vs. female	1.10	1.00–1.22	0.059
Any allergic disease	Middle SES vs. low SES	1.12	0.99–1.27	0.072
Any allergic disease	High SES vs. low SES	1.26	1.12–1.43	<0.001
Asthma	Male vs. female	1.30	1.06–1.58	0.011
Allergic rhinitis	Male vs. female	1.12	0.95–1.31	0.182
Allergic conjunctivitis	Male vs. female	1.00	0.80–1.24	1.000
Atopic dermatitis	Male vs. female	1.09	0.95–1.25	0.246
Food allergy	Male vs. female	Not reported consistently	Not reported consistently	0.182

ORs are unadjusted odds ratios calculated from aggregate counts reported in the manuscript tables. SES, socioeconomic status; CI, confidence interval. Because individual-level data were not available, multivariable adjusted odds ratios could not be calculated.

## 4 Discussion

In this nationwide study of children aged 0–5 years, we identified distinct demographic and clinical patterns among pediatric patients diagnosed with migraine who also had early-childhood allergic diseases. We demonstrated that allergic comorbidity is associated with a younger age of migraine diagnosis and with specific demographic characteristics, whereas sex differences were minimal and largely confined to asthma. These are novel insights into the intersection between early-life atopy and migraine.

Several studies have demonstrated that atopic diseases are associated with increased migraine risk in the pediatric population. Wei et al. (17) examined the relationship between antecedent allergic diseases (including atopic dermatitis, allergic conjunctivitis, allergic rhinitis, and asthma) and the subsequent risk of migraine among 16,130 individuals aged 7–18 years who were diagnosed with migraine and 64,520 matched controls without a history of migraine. Children with preceding allergic diseases had a significantly higher risk of migraine compared with controls. Allergic rhinitis demonstrated the highest adjusted odds ratios (aORs) (aOR: 2.17; 95% CI: 2.09–2.26). Children diagnosed with all four allergic diseases had the greatest risk, with an aOR of 3.59 (95% CI: 2.91–4.44). Moreover, they found that the subsequent risk of migraine was associated with a cumulative effect of concurrent allergic diseases and the extent of allergy-related healthcare utilization. The authors concluded that children with allergic diseases are at increased risk for subsequent migraine during school age (17). Our research findings align with those of Wei et al., as both studies present a meaningful association between allergic diseases and migraine during childhood. In both cohorts, allergic comorbidities were common among children with migraine, and the existence of multiple allergic conditions appeared to contribute to a greater overall disease burden. Our results are similar to the cumulative pattern reported by Wei et al., which demonstrates that allergic diseases cluster within children and vary across demographic subgroups, including age and sex. Moreover, both studies highlight that specific allergic disorders, particularly atopic dermatitis, allergic rhinitis, and asthma, play a prominent role in the clinical profile of children diagnosed with migraine.

Furthermore, both our study and that of Wei et al. specifically evaluated children whose allergic diseases were diagnosed before migraine onset, establishing temporal precedence and reducing surveillance bias. This prospective sequence reduces the likelihood that the association is driven solely by differential healthcare utilization and strengthens the inference of a true underlying relationship.

Fuxench et al. (37) evaluated the risk of headache and migraine among children and adults with atopic dermatitis. Their study included 409,431 children with atopic dermatitis and 1,809,029 matched control children. Compared with controls, children with atopic dermatitis exhibited a 5% increased risk of migraine [hazard ratio (HR) 1.05, 95% CI: 1.03–1.08] and a 10% increased risk of headaches (HR: 1.10, 95% CI: 1.09–1.12) (37). Our findings are consistent with those of Fuxench et al., who similarly demonstrated an elevated risk of migraine among children with atopic dermatitis. While the large population-based cohort of Fuxench et al. showed a modest but statistically significant increase in migraine and headache risk among children with atopic dermatitis, our study identified atopic dermatitis as the most prevalent allergic comorbidity among children with migraine. Therefore, both studies support a link between allergic disease, specifically atopic dermatitis, and migraine in the pediatric population. However, Fuxench et al. quantified the longitudinal risk of developing migraine among children and adults with atopic dermatitis, whereas our study characterized the demographic and clinical parameters of children already diagnosed with migraine, revealing age-related patterns, sex distributions, and socioeconomic differences across allergic phenotypes. These complementary findings reinforce the growing evidence that a variety of allergic diseases, including atopic dermatitis, contribute to migraine susceptibility and may share underlying inflammatory or immunologic mechanisms.

Wang et al. (19) evaluated the risk of migraine among 309,138 children with allergic conjunctivitis and an equal number of matched controls without allergic conjunctivitis. The incidence of migraine was 1.92-fold higher in the allergic conjunctivitis cohort than in controls. The risk was particularly elevated among boys and among children younger than 6 years. The risk of migraine increased with longer follow-up and peaked 4–5 years after the diagnosis of allergic conjunctivitis (19). Our findings correspond with those of Wang et al., demonstrating a meaningful association between allergic disease and migraine throughout childhood, although the two studies investigate this relationship from different methodological perspectives. In our cohort, allergic conjunctivitis is one of several allergic comorbidities among children with migraine, with affected patients presenting at a slightly older median age and demonstrating characteristic demographic patterns across age groups, sex, and socioeconomic status. Similarly, Wang et al. reported a significantly higher incidence of subsequent migraine among children with allergic conjunctivitis, with the risk particularly elevated in boys and in younger children, which are patterns that are consistent with the male predominance and age-related distributions observed in our allergic subgroups. Our study provides a cross-sectional characterization of allergic diseases in children who are later diagnosed with migraine, whereas Wang et al. present longitudinal evidence indicating that allergic conjunctivitis confers an increased and time-dependent risk of developing migraine, with risk peaking several years after the initial allergic diagnosis.

 Asthma was the only allergic condition in our cohort that demonstrated a significant sex difference, being more common in males. It has been reported that asthma is more common among males during childhood, as they have higher blood eosinophil counts, fractional exhaled nitric oxide, total and allergen-specific IgE, and periostin levels than females. Sex hormones play a key role in immune modulation, as estrogen and progesterone promote type 2 inflammation while testosterone exerts suppressive effects. This provides a mechanistic explanation for the male predominance of childhood asthma (38). The persistence of this pattern within a migraine cohort suggests that asthma may represent a particularly sex-sensitive allergic phenotype in relation to migraine during early childhood.

In our cohort, the majority of children with allergic diseases, excluding those with asthma, belonged to the high SES. The relationship between SES and allergic diseases is multifaceted and varies by condition. Atopic dermatitis is more commonly reported in higher socioeconomic groups in European studies and among infants, though this association is inconsistent globally (39). Allergic rhinoconjunctivitis was also reported to be significantly more prevalent in higher socioeconomic groups (40). Asthma disproportionately affects socioeconomically disadvantaged and racial/ethnic minority populations due to environmental exposures (including indoor allergens, air pollution, smoke, and poor housing conditions (41). In our study, most children with migraine were from lower SES; however, the majority of children diagnosed with both migraine and allergic disease(s) belonged to higher SES. Evidence indicates that lower SES is associated with a higher prevalence of migraine and greater disease burden in pediatric populations, although this relationship is complex and varies by region (42). These patterns underscore the importance of considering social determinants of health when evaluating allergic comorbidity in pediatric migraine.

Multimorbidity was relatively uncommon in our study, a finding that is expected given the participants' young age. The atopic march typically unfolds over several years, and the likelihood of multiple concurrent allergic diagnoses increases later in childhood (43, 44). Nevertheless, even a single allergic condition in early childhood may reflect underlying immune dysregulation that might be relevant to migraine susceptibility.

In our study, we demonstrated that the presence of allergic disease in early childhood may serve as a clinical indicator of earlier migraine onset. We suggest that pediatricians, pediatric neurologists, and family physicians consider incorporating structured allergy assessments for young children diagnosed with migraine, particularly those with atopic dermatitis, food allergy, or asthma. Although current evidence is limited regarding whether improved management of allergic disease can modify migraine trajectory, reducing systemic inflammation and allergen exposure may offer a potential benefit.

A major strength of this study is its real-world, large, nationwide sample, long observation period, and focus on allergic diseases during early childhood. The scale and completeness of the database confer substantial statistical power. Nevertheless, several limitations should be acknowledged. First, the study relied on retrospective electronic medical record data, restricting analyses to information documented for clinical and not for research purposes. This may have resulted in an underestimation of both migraine cases and allergic diseases that did not lead to medical evaluation. Second, the dataset lacked detailed migraine phenotyping, including headache frequency, severity, aura status, functional disability measures, drug therapy, and treatment response. Therefore, these limited our ability to characterize disease heterogeneity and to determine unequivocally the association between allergic diseases and migraine. Third, important allergy-related clinical and laboratory parameters, such as symptom severity, skin prick tests, serum IgE concentrations, eosinophil counts, and pulmonary function tests, were not available, constraining the characterization of allergic diseases. Fourth, potential confounders, including environmental exposures, family history of allergic disorders and migraine, additional comorbidities, and healthcare utilization patterns, were not included and therefore could not be addressed. Fifth, the study did not include a control group of children with allergic diseases who did not have migraine, limiting our ability to determine whether the observed associations are specific to children with both conditions or reflect broader characteristics of the allergic pediatric population. Sixth, Healthcare utilization could not be evaluated from the available aggregate data; therefore, surveillance intensity remains a potential unmeasured confounder in this retrospective database analysis.

## 5 Conclusion

This study demonstrates that allergic diseases in early childhood are associated with younger age at migraine diagnosis and with distinct demographic patterns. The distribution of allergic phenotypes observed in early-life in the cohort may play a role in migraine susceptibility. Future research should explore the mechanistic pathways linking early-childhood atopy and migraine and evaluate whether targeted management of allergic disease can modify migraine outcomes in young children.

## Data Availability

The datasets presented in this article are not readily available because, according to Israeli law, the data cannot be shared. Requests to access the datasets should be directed to shine6@ walla.co.il.
